# Metachronous nodal metastases from HPV-associated penile carcinoma in situ

**DOI:** 10.1016/j.eucr.2025.103183

**Published:** 2025-09-01

**Authors:** Ryan Antar, Brij Kathuria, Michael Wynne, Megan Clyne, Andrew Hall, Daniel Stein, Michael Whalen

**Affiliations:** a Department of Urology, George Washington University School of Medicine, Washington, DC, USA; b Department of Pathology, George Washington University School of Medicine, Washington, DC, USA; c Department of Urology, Georgetown University School of Medicine, Washington, DC, USA

## Abstract

Penile intraepithelial neoplasia (PeIN) is a premalignant precursor of squamous cell carcinoma (SCC) with a 5–15 % risk of progression. Nodal metastasis without histologic invasion is exceedingly rare. We describe a 40-year-old man with recurrent, HPV-positive basaloid PeIN of the glans and distal urethra who developed right inguinal and pelvic nodal metastases despite serial resections consistently showing carcinoma in situ. Genomic profiling revealed EGFR and JUN amplifications with NCOR1 and PRKAR1A losses, alterations linked to aggressive tumor biology. This case highlights the paradoxical metastatic potential of high-grade PeIN and underscores the need for molecular risk stratification in surveillance and management.

## 1 Introduction

PeIN is a rare premalignant precursor of penile SCC, with an estimated 5–15 % risk of progression.^1^^,^^2^ High-risk HPV, particularly HPV-16, is present in most PeIN lesions and roughly one-third of invasive penile cancers.^3^ Additional risk factors include lack of circumcision, chronic inflammatory dermatoses, prior penile surgery, and immunosuppression.^4^ Diagnosis is often delayed due to overlapping clinical features with benign inflammatory conditions, an asymptomatic course, and infrequent follow-up.^4^ By definition, PeIN is confined to the epithelium, making nodal or systemic metastasis, whether synchronously or even metachronously, highly unlikely and biologically perplexing. However, isolated case reports have described nodal metastases without histologic invasion, challenging the traditional stepwise model of tumor progression. We report a rare case of recurrent high-grade HPV-related PeIN with extensive nodal metastases and explore hypothesis-generating mechanisms and molecular features that may explain this paradoxical presentation.

### 1.1 Case presentation

**Fig. 1:**
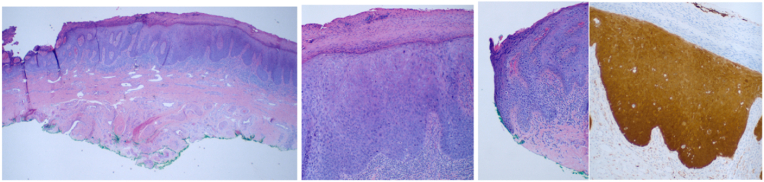
Sections show PeIN/HSIL extending to the inked peripheral margin. The lesion shows the full thickness of the squamous epithelium to be atypical, with mitotic activity in the upper third. The interface with the stroma is smooth with no evidence of invasion. Immunostain for p16 (right) shows block-like positivity consistent with HPV related PeIN/HSIL.

**Fig. 2:**
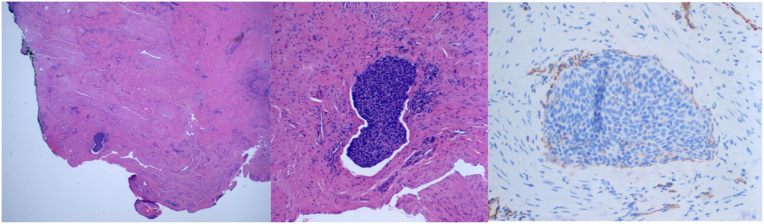
Sections of second distal urethral biopsy showed basaloid tumor cells present within a lymphatic space. Immunostaining for CD31 highlights the endothelial cells of the lymphatic space, which contains the tumor.

**Fig. 3:**
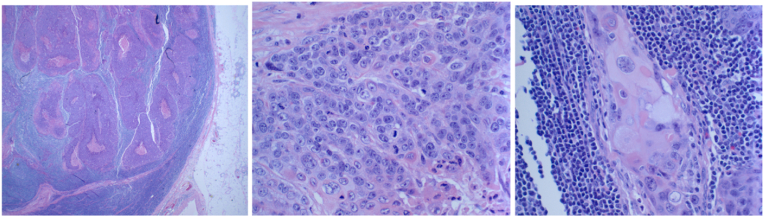
Sections of right superficial lymph node show involvement by squamous cell carcinoma with predominantly basaloid features, accompanied by some areas of keratinization.

A 40-year-old male was initially referred in 2017 for a peri-meatal lesion located on the ventral glans penis. Biopsy revealed squamous cell carcinoma in situ (CIS), and underwent wide local excision with first-stage Johansson urethroplasty two months later. Pathology revealed positive margins (superior, right, and left lateral). A repeat resection demonstrated a CIS recurrence with extension to the medial and superior margins nearly eight months after the initial procedure. He was subsequently treated with topical 5-fluorouracil cream in early 2018 but discontinued due to severe dermatitis. In May 2021, retrograde urethrogram demonstrated a distal urethral stricture, and was lost to follow-up. He re-presented in 2022 and underwent partial glans resection with complex reconstruction. Urethral biopsies at the time demonstrated high-grade PeIN with positive margins between the 9 o'clock and 12 o'clock positions and the 1 o'clock and 3 o'clock positions of the right and left glans specimens, respectively (Fig. 1). A surveillance cystoscopy and cytology in July 2023 were negative. However, in October 2023, the patient developed gross hematuria, and cytology was positive for malignant squamous cells. CT abdomen/pelvis and chest revealed bilateral nephrolithiasis, a 3 mm lingular pulmonary nodule, and no inguinal, pelvic, or retroperitoneal lymphadenopathy. In January 2024, he underwent repeat first-stage Johansson urethroplasty, excision of the urethral plate, and wide excision of the penile lesion with complex glans reconstruction. However, pathology again revealed extensive high-grade squamous intraepithelial neoplasia (HSIL) across multiple margins, including the distal, lateral, and urethral resection sites. Notably, HSIL and areas suspicious of lymphatic invasion were observed on permanent but not frozen sections. Five months later, erythema of the glans prompted excisional biopsy and cystoscopy, which revealed recurrent HSIL involving both the penile lesion and urethral specimens, extending to the inked margin. By January 2025, a palpable right inguinal lymph node was detected; surveillance CT revealed a 2.7 cm node, and nodal biopsy confirmed HPV-positive basaloid squamous cell carcinoma (p16 and p40 positive). He subsequently underwent bilateral superficial and deep inguinal lymph node dissection in concert with repeat distal urethrectomy. The final pathology from the lymph node dissection showed 4/10 positive superficial right inguinal nodes (largest 2.4 cm) and 1/1 positive deep right node (0.4 cm), all without extranodal extension. All left-sided superficial (0/11) and deep (0/1) lymph nodes were negative; total bilateral nodal yield was 23. The biopsy specimen from the dorsal urethra was negative for tumor on the frozen section; however, it demonstrated a high-grade squamous intraepithelial lesion (HSIL) on the permanent section (Fig. 2, Fig. 3). Clinical staging was consistent with pTisN2M0R1. Given the patient's pathology findings, which revealed a total of 5 positive nodes, the patient returned to the operating room in March 2025 for a robotic bilateral pelvic lymph node dissection (BPLND). The template included internal iliac, external iliac, obturator, deep obturator, and common iliac lymph nodes. Final pathology revealed metastatic SCC in 4 of 24 right pelvic nodes, with the largest node measuring 3.0 cm with no extranodal extension. An additional node of Cloquet and a deep obturator node on the right side were positive for metastatic SCC with measurements of 3.0 cm and 1.0 cm, respectively. A PET scan performed in May 2025 revealed hypermetabolism in bilateral external iliac nodes, with greater left-sided activity, though findings were equivocal given prior negative nodes. Upon final pathology from BPLND, his pathological staging was upgraded to pTisN3(6/24)M0R1. Comprehensive genomic profiling (Tempus xT) confirmed HPV-driven oncogenesis with >100 HPV reads. Somatic mutations included EGFR and JUN amplifications and NCOR1 and PRKAR1A losses. Variants of uncertain significance were detected in SUFU, PRDM1, CARD11, TSC1, and MBD4. Additionally, testing revealed low tumor mutational burden (5.3/Mb). Our patient's mismatch repair gene expression was intact (MLH1, PMS2, MSH2, MSH6). With a low PD-L1 combined positive score and negative HER2 status, the patient was not a candidate for targeted immunotherapy. Serial Natera Signatera ctDNA assays demonstrated a rise in mean tumor molecules from 0.15 to 0.31 MTM/mL over two weeks, consistent with residual disease.

Based on NCCN guidelines for node-positive penile squamous cell carcinoma, the patient was started on adjuvant chemotherapy with a platinum-based regimen. He is receiving four cycles of TIP (paclitaxel, ifosfamide, and cisplatin), the recommended first-line protocol for N3 disease without extranodal extension or visceral metastasis. The patient has deferred radiation therapy at this time.

## 2 Discussion

Nodal or systemic spread from penile CIS is exceedingly rare, with only a few cases reported. Avrach and Christensen (1976) described erythroplasia of Queyrat with inguinal node metastases treated by circumcision and node dissection, later progressing to disseminated disease.^5^ Eng et al. (1995) reported CIS managed with circumcision and topical 5-fluorouracil, which recurred years later with inguinal and para-aortic metastases, following chemotherapy and lymphadenectomy, the patient remained disease-free at three years.^6^ Kim et al. described a 51-year-old man with glanular CIS and synchronous bulky bilateral inguinal metastases who underwent partial penectomy with bilateral inguinal and pelvic lymphadenectomy (pTisN3). He received adjuvant platinum-based chemotherapy and pelvic radiation and remained disease-free at 6 months.^7^

Despite repeated resections showing only intraepithelial disease, our patient developed nodal metastases without an identifiable invasive component. Prior studies highlight the prognostic significance of PeIN-positive margins. In a study of 378 men undergoing penile-sparing surgery, local recurrence occurred in 18.6 % with PeIN at the margin versus 9.4 % with negative margins. However, no nodal recurrences were seen during a median 31 months of follow-up. After adjusting for T stage and grade, a positive margin was associated with a statistically significantly higher risk of local recurrence.^8^ Contemporary data from a single-institution cohort of 18 patients with PeIN-positive margins following organ-sparing surgery reported high recurrence rates across management strategies (i.e., adjuvant topical therapy, repeat surgical resection, etc.). Among those observed, 36.4 % developed recurrence, including three local, two regional, and one systemic. Re-resection carried a 50 % recurrence rate, with one patient progressing to systemic disease and dying 22 months postoperatively.^9^ These findings highlight PeIN-positive margins as a clinically significant predictor of recurrence and progression, even without evident invasion, and establish precedent for regional and distant spread. Our patient's presentation defies traditional expectations of in situ disease and raises questions about the mechanisms underlying early metastasis. This paradox has prompted several hypotheses, many of which draw from similar patterns observed in other cancer types, where metastasis may precede apparent histologic invasion.

The simplest plausibility is that a focus of invasive carcinoma was present but not identified on histopathology. Even with thorough sampling, tiny foci of invasion can be missed (“occult” invasion). In analogous cases of breast carcinoma in situ with axillary nodal metastases, investigators postulated that random tissue sampling might miss a small invasive component.^10^ Ozzello and Sampitak demonstrated by ultrastructural examination that areas appearing as only CIS on light microscopy can harbor microscopic invasion when examined at higher resolution,^11^ a concept corroborated in a study of serous endometrial carcinoma in situ (SEIC) and superficial serous endometrial SCC, where occult nodal metastasis was ultimately discovered despite an initial in-situ diagnosis. Yoshioka et al. reported that 4 of 17 SEIC/SSC patients who underwent pelvic/para-aortic lymph node dissection were found to have nodal metastases.^12^

Although the entire lesion from our patient was submitted for histologic analysis, an undetected microinvasive focus (perhaps destroyed during biopsy or present in unsampled tissue) cannot be excluded. However, its likelihood is reduced by multiple resections. Similar diagnostic “blind spots” have been reported in melanoma. One study described a melanoma in situ that produced a nodal metastasis seven years after excision, and re-review showed that nearly one-third of such cases harbored overlooked microinvasive nests detectable only with more sensitive immunohistochemistry.^13^ Additionally, repeated urethral and glans resections in our case may have been a potential etiology of lymphatic seeding and spreading.

Another possibility is that an invasive component briefly arose and then regressed (due to an immune response or other factors), leaving only intraepithelial disease behind. In our case, initial CIS and its recurrence were treated surgically and with topical therapy. Yet, years later, the penile lesion again showed only intraepithelial disease while nodal metastases were confirmed. This suggests a transient invasive clone may have seeded the lymphatics before regressing, which is a “vanishing primary” phenomenon described in melanoma and breast cancer, where metastatic disease occurs with little or no residual primary tumor. In penile cancer, the spontaneous regression of the primary lesion has not been documented. Still, an aggressive host immune response (perhaps augmented by our young patient's prior HPV exposure) might theoretically eradicate invasive cells in the penis after metastasis had occurred.

The basaloid subtype of penile SCC is recognized for its aggressive behavior and early metastatic potential. According to the “seed and soil” model, certain tumor subclones may preferentially thrive in specific microenvironments. In our case, it is conceivable that a small population of basaloid dysplastic cells acquired the capacity to breach the basement membrane, intravasate, and colonize inguinal nodes before overt invasion was histologically evident. Once established, these metastatic clones may have proliferated more aggressively than the superficially confined primary lesion. HPV-related oncogenic changes may have further facilitated earlier than expected dissemination and survival in the nodal niche.

Emerging evidence on circulating tumor cells (CTC) suggests that metastases may occur even at the in situ stage. CTCs have been detected in patients with ductal carcinoma in situ of the breast, suggesting that cells may disseminate before a tumor is overtly invasive, at least with our current diagnostic capabilities.^14^ The “parallel progression” model further proposes that early neoplastic cells may generate metastases along an accelerated, invasion-independent pathway.^15^ While intriguing and hypothesis-generating, these theories remain unproven in penile cancer, let alone in many other cancers.

Genomic profiling in our patient revealed alterations plausibly linked to aggressive tumor biology. EGFR amplification has been implicated in squamous carcinogenesis across HPV-associated head and neck and anogenital cancers. JUN amplification, encoding the AP-1 transcription factor c-Jun, drives proliferation, survival, angiogenesis, and epithelial–mesenchymal transition; in oral SCC, c-Jun overexpression correlates with nodal metastasis and aggressive phenotypes, particularly with HPV infection and tobacco exposure.^16^ Loss of NCOR1 has been associated with poorly differentiated phenotypes in head and neck SCC,^17^ while PRKAR1A inactivation has been reported in advanced, poorly differentiated melanomas with frequent regional and distant spread.^18^ Although classically a Hedgehog pathway suppressor, SUFU has paradoxically been shown to be overexpressed in cervical SCC.^19^ Additional variants of uncertain significance were identified in PRDM1, CARD11, TSC1, and MBD4, genes linked to oncogenesis, immune regulation, or genomic stability, though their contribution in this setting remains unclear. Collectively, these findings highlight the molecular heterogeneity that may underlie the patient's aggressive clinical course.

## 3 Conclusion

Our case highlights that penile CIS, though histologically confined to the epithelium, can harbor metachronous metastatic potential. The progression from repeated intraepithelial resections to nodal metastases underscores the malignant potential of in situ lesions and the importance of vigilant surveillance, which currently relies on physical examination and imaging, and will likely evolve to incorporate periodic ctDNA measurements. Clinicians should maintain a high index of suspicion in high-grade penile lesions and ensure thorough nodal assessment, even in the absence of overt signs of invasion.

## CRediT authorship contribution statement

**Ryan Antar:** Conceptualization, Investigation, Methodology, Writing – original draft, Writing – review & editing. **Brij Kathuria:** Writing – original draft, Writing – review & editing. **Michael Wynne:** Investigation, Writing – original draft, Writing – review & editing. **Megan Clyne:** Writing – review & editing. **Andrew Hall:** Writing – original draft, Writing – review & editing. **Daniel Stein:** Writing – original draft, Writing – review & editing. **Michael Whalen:** Conceptualization, Investigation, Methodology, Project administration, Supervision, Writing – original draft, Writing – review & editing.

## Informed consent

Patient discussed in case presentation provided written informed consent to participate in the study.

## Data availability statement

Data sharing is not applicable to this article since no datasets were produced or analyzed in the current study. All data supporting this study are available in the paper.

## Funding

This research did not receive any specific grant from funding agencies in the public, commercial, or not-for-profit sectors.

## Declaration of competing interest

The authors declare that they have no known competing financial interests or personal relationships that could have appeared to influence the work reported in this paper.
